# Association between triglyceride-glucose index and sarcopenia in patients with chronic kidney disease

**DOI:** 10.3389/fendo.2025.1626241

**Published:** 2025-09-18

**Authors:** Yifen Zhang, Fan Zhang, Wenjian Li

**Affiliations:** ^1^ Department of Endocrinology, Changzhou Third People’s Hospital, Changzhou, China; ^2^ Department of Clinical Nutrition, Changzhou Third People’s Hospital, Changzhou, China; ^3^ Department of Urology, Changzhou Third People’s Hospital, Changzhou, China; ^4^ Changzhou Clinical College, Xuzhou Medical University, Changzhou, China

**Keywords:** chronic kidney disease, sarcopenia, triglyceride-glucose index, insulin resistance, cross-sectional study

## Abstract

**Objective:**

This study aimed to examine the relationship between the triglyceride-glucose index (TyG) and sarcopenia in patients with chronic kidney disease (CKD). The aim was to gain new insights into preventing and treating sarcopenia in CKD patients.

**Methods:**

The study utilized data from two cohorts, including the NHANES 2011–2018 cohort in the United States and the 2018–2023 cohort in China. After applying uniform inclusion and exclusion criteria, 827 patients with CKD in the US cohort and 1,038 patients with CKD in the Chinese cohort were ultimately included in the study. The relationship between the TyG index and sarcopenia was analyzed using logistic regression modeling and multivariate adjustment. The dose-response relationship was also explored using restricted cubic spline (RCS) modeling. Subgroup analyses were also conducted to investigate the potential heterogeneity among different characteristic subgroups.

**Results:**

The TyG index was found to be significantly and positively associated with sarcopenia in patients with CKD in both the United States and Chinese cohorts. In the US cohort, the risk of sarcopenia was increased 4.01-fold in the highest TyG quartile group compared with the lowest quartile group (P=0.002). In the Chinese cohort, the corresponding risk was increased 3.25-fold (P<0.001). Furthermore, the RCS analysis corroborated the nonlinear positive association. Subgroup analyses revealed that the correlation between TyG and sarcopenia was more pronounced in patients without diabetes and without metabolic syndrome.

**Conclusion:**

The TyG index may serve as a potential biomarker for assessing sarcopenia in CKD patients, thereby supporting the critical role of insulin resistance in developing sarcopenia. Further research is required to elucidate the precise mechanisms by which TyG is associated with sarcopenia and to develop tailored intervention strategies for different patient groups.

## 1 Introduction

Chronic kidney disease (CKD) is a progressive pathological state that is defined by a continuous decline in renal function. This may eventually result in the development of end-stage renal disease (ESRD) (1, 2). The latest data indicate that the global incidence of CKD is approximately 8% to 14%, with an increasing trend over time. This has significant economic and mental health implications for patients and society (2–4). CKD affects not only the structure and function of the kidney but also is associated with a variety of metabolic disorders and complications, including cardiovascular disease, anemia, bone disease, and muscle atrophy (2, 5–7).

Sarcopenia is a disorder that is characterized by a reduction in skeletal muscle mass and strength (8, 9). In patients with CKD, the prevalence of sarcopenia is significantly increased by a combination of factors, including long-term renal impairment, which leads to the accumulation of metabolic wastes and toxins in the body, as well as nutritional deficiencies and inflammatory responses (10, 11). Sarcopenia not only affects the quality of life of patients but may also result in a reduction in exercise capacity, an increased risk of falls, a decline in quality of life, and an unfavorable prognosis, thereby posing a significant threat to the life and health of patients (10, 12).

In recent years, the triglyceride-glucose index (TyG), as a convenient and easily accessible metabolic assessment tool, has been widely employed in the quantitative evaluation of insulin resistance and metabolic disorder status. The clinical convenience of this approach is evident in its reliance on routine testing of fasting triglycerides (TG) and fasting plasma glucose (FPG) data, thereby obviating the need for complex technical procedures, such as the gold standard but cumbersome high insulin-euglycemic clamp technique (13). The existing literature indicates that the TyG index is associated with the onset and progression of a range of chronic diseases, including type 2 diabetes, non-alcoholic fatty liver disease, and cardiovascular disease (14–16). Moreover, preliminary research has indicated a potential correlation between the TyG index and sarcopenia (17, 18). Of particular significance are its muscle metabolism-related properties, as elevated TyG directly reflects dysfunction in the muscle insulin signaling pathway caused by lipotoxicity (elevated TG) and glucotoxicity (elevated FPG). These properties closely align with the core pathophysiological mechanisms of sarcopenia (decreased protein synthesis and increased protein breakdown) (19, 20). These characteristics render TyG particularly well-suited for assessing sarcopenia risk in patients with CKD, as this demographic necessitates frequent monitoring of lipid and glucose levels, and TyG can be seamlessly integrated into routine clinical management.

Although the association between the TyG index and sarcopenia in the general population has been confirmed, its role in patients with CKD remains unclear. This population faces a significantly elevated risk of sarcopenia due to the overlapping effects of insulin resistance, chronic inflammation, and metabolic disorders. Consequently, there is a necessity to explore efficient risk markers. To this end, this study employs a China-U.S. dual-cohort design, which is unique in this context, to validate the association between the TyG index and sarcopenia in CKD patients and assess its potential as a risk stratification tool for insulin resistance.

## 2 Materials and methods

### 2.1 Study population

**Figure 1:**
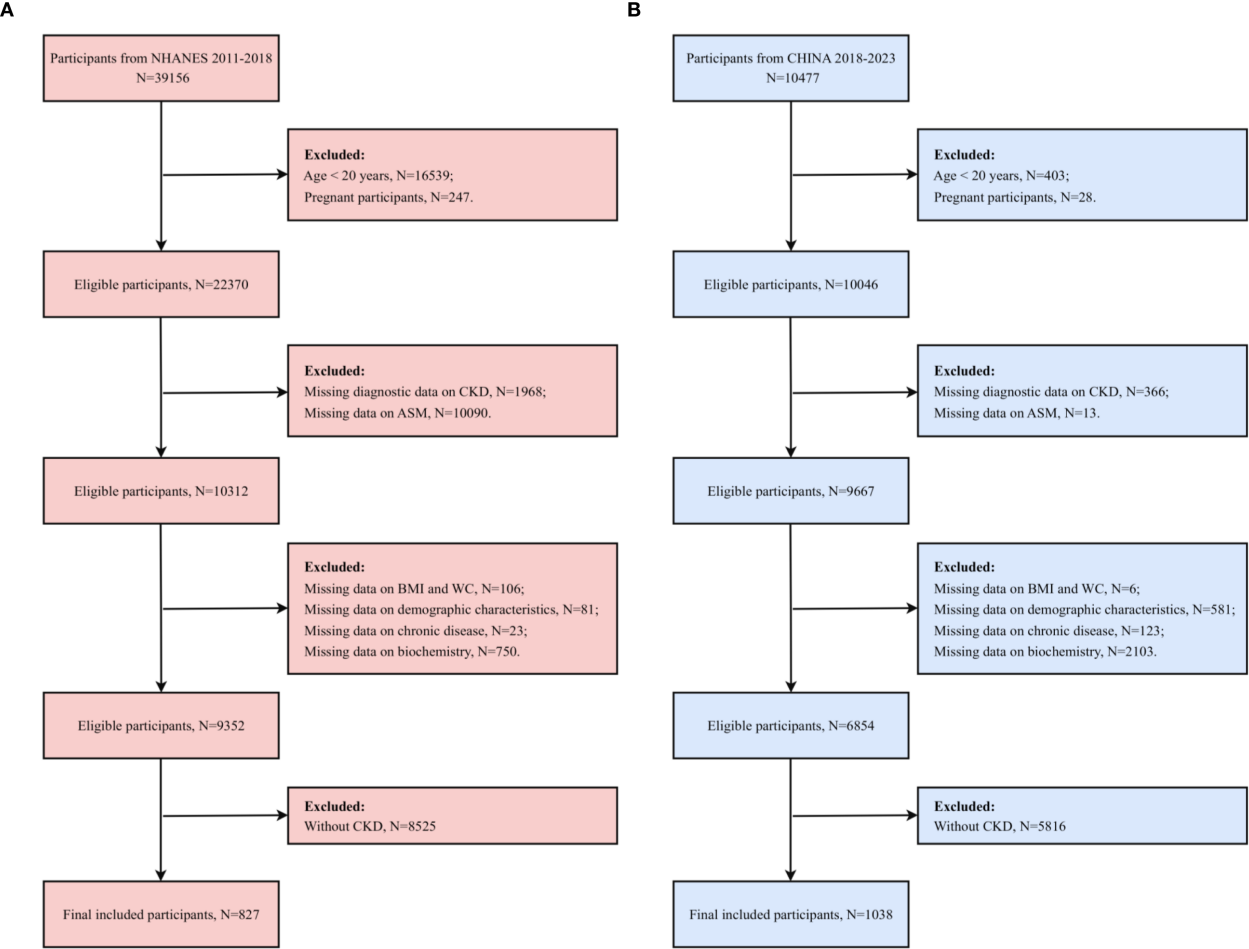
Participant screening flowchart. **(A)** the US cohort; **(B)** the Chinese cohort.

The present study employed a cross-national dual-cohort design, incorporating data from the NHANES from 2011 to 2018 and the clinical database of the Changzhou Third People’s Hospital, China, from 2018 to 2023. The original data set of the US cohort comprised 39,156 participants, derived from four consecutive cycles of the NHANES cross-sectional survey. This study design was approved by the Ethics Review Board of the National Center for Health Statistics (NCHS), with all participants providing written informed consent. The study was deemed exempt from secondary ethical review of the NHANES data by the Ethics Committee of Changzhou Third People’s Hospital, based on the ethical guidelines of the Declaration of Helsinki and the public nature of the NHANES data. The protocol of the Chinese cohort study was formally reviewed and approved by the Ethics Committee of Changzhou Third People’s Hospital. The Chinese cohort data utilized in this study were obtained from outpatient patients undergoing routine follow-up at Changzhou Third People’s Hospital between 2018 and 2023. All participants were in the outpatient routine management phase, except those who had experienced acute hospitalization or critical illness. The Chinese cohort included 10,477 participants who underwent a nutritional assessment, and after screening with uniform exclusion criteria (age< 20 years, pregnancy, missing key data, and non-CKD patients), 827 cases were finally retained for the US cohort, and 1,038 eligible samples were retained for the Chinese cohort (**Figure 1**).

### 2.2 Assessment of CKD

To assess the state of renal function, the urine albumin-to-creatinine ratio (UACR) and estimated glomerular filtration rate (eGFR) were employed as the core assessment indexes in this study. The eGFR was calculated using the Chronic Kidney Disease Epidemiology Collaboration (CKD-EPI) formula (21). The diagnosis of CKD was made in strict accordance with international consensus criteria based on a combined assessment of the UACR and the eGFR. Specifically, a UACR of at least 30 mg/g and/or an eGFR of less than 60 mL/min/1.73m² was defined as CKD (9). Patients were classified into five stages of CKD based on disease severity, according to the Kidney Disease Quality Outcome Initiative (K/DOQI) CKD classification. Stage 1: eGFR ≥ 90 mL/min/1.73m^2^; Stage 2: eGFR 60–89 mL/min/1.73m^2^; Stage 3: eGFR 30–59 mL/min/1.73m^2^; Stage 4: eGFR 15–29 mL/min/1.73m^2^; Stage 5: eGFR< 15 mL/min/1.73m^2^ (22).

### 2.3 Assessment of sarcopenia

The appendicular skeletal muscle mass (ASM) of the extremities was measured using dual-energy X-ray absorptiometry (DXA) in the US cohort and bioelectrical impedance analysis (BIA) in the Chinese cohort, respectively. Standardized assessment was performed by calculating the extremity skeletal muscle mass index (ASMI=ASM/BMI). The diagnostic thresholds for sarcopenia were referenced in the guidelines of the Foundation for the National Institutes of Health (FNIH). Specifically, the ASMI was required to be less than 0.789 for males and less than 0.512 for females to be considered sarcopenia (23).

### 2.4 Assessment of the TyG index

TG and FPG testing was completed by a professional team after subjects had fasted for at least 8 hours and was measured using a fully automated biochemical analyzer. The TyG index was calculated using the following formula (13):


TyG= ln[TG (mg/dL) × FPG (mg/dL)/2]

### 2.5 Assessment of covariates

To control for potential confounders, a multivariate correction model was constructed to include covariates such as gender, age, marital status (cohabitation and solitude), smoking, alcohol consumption, and chronic diseases (diabetes, hypertension, and metabolic syndrome). Diabetes mellitus (DM) was defined as having been diagnosed by a medical professional with an FPG level of ≥126 mg/dL or glycosylated hemoglobin (HbA1c) level of ≥6.5% or treatment with glucose-lowering medication; hypertension was defined as a self-reported history of the disease or use of antihypertensive medication; and metabolic syndrome was diagnosed when participants met at least three of the following five criteria (1): TG levels of no less than 150 mg/dL (2); high-density lipoprotein cholesterol (HDL-c) levels of less than 40 mg/dL (men) or 50 mg/dL (women) (3). FPG levels of 110 mg/dL or more (4); WC of more than 102 cm (men) or 88 cm (women) (5); Systolic blood pressure of more than 130 mmHg or diastolic blood pressure of more than 85 mmHg, or both (24).

### 2.6 Statistical analysis

Continuous variables were expressed as median (interquartile range), and comparisons between groups were made using the Mann-Whitney U test. Categorical variables were described in terms of frequency and percentage, and differences between groups were assessed using the chi-square test. Multi-model logistic regression was used to analyze the association between the TyG index and sarcopenia. Model 1 was unadjusted for any variables, Model 2 was adjusted for age and gender, and Model 3 was further adjusted for marital status, smoking, alcohol consumption, comorbidity status, and eGFR. The dose-response relationship between the TyG index and sarcopenia was explored using restricted cubic spline (RCS) modeling, and subgroup analyses and interaction tests were performed based on variables such as gender and comorbidity status. Statistical significance was defined as a two-sided P value of less than 0.05, and all analyses were conducted using R 4.4.0, with SPSS 23.0, and data visualization performed using GraphPad Prism 9.0.

## 3 Results

### 3.1 Baseline characteristics of CKD patients with and without sarcopenia in the US cohort

In this study, a total of 827 patients with CKD from a US cohort were analyzed, comprising 711 individuals without sarcopenia and 116 patients with sarcopenia. The results demonstrated that patients in the sarcopenia group exhibited a markedly higher proportion of males than those in the non-sarcopenia group (62.93% vs. 40.08%, P< 0.001) and were older (50.00 years vs. 45.00 years, P< 0.001). No significant differences between the two groups were observed in marital status, smoking, or drinking habits (P > 0.05). However, the prevalence of DM (43.10% vs. 29.11%, P=0.003) and metabolic syndrome (54.31% vs. 41.63%, P=0.011) was significantly higher in the sarcopenia group than in the non-sarcopenia group. Furthermore, patients in the sarcopenia group exhibited significantly elevated BMI, WC, FPG, HbA1c, total cholesterol (TC), TG, and uric acid levels compared to those in the non-sarcopenia group. Conversely, the HDL-c levels were diminished (P< 0.05). No significant differences were observed in blood creatinine, blood urea nitrogen (BUN), and eGFR between the two groups (P > 0.05). Notably, the TyG index was significantly higher in the sarcopenia group (P< 0.001) (**Table 1**).

**Table 1:** Baseline characteristics of CKD patients with and without sarcopenia in the US cohort.

Variables	Total (n=827)	Non-sarcopenia (n=711)	Sarcopenia (n=116)	*P*
Gender, n (%)				<0.001
Male	358 (43.29)	285 (40.08)	73 (62.93)	
Female	469 (56.71)	426 (59.92)	43 (37.07)	
Age (years)	46.00 (35.00, 53.00)	45.00 (34.00, 52.00)	50.00 (38.00, 54.00)	<0.001
Marital Status, n (%)				0.162
Cohabitation	470 (56.83)	411 (57.81)	59 (50.86)	
Solitude	357 (43.17)	300 (42.19)	57 (49.14)	
Smoking, n (%)				0.936
Yes	375 (45.34)	322 (45.29)	53 (45.69)	
No	452 (54.66)	389 (54.71)	63 (54.31)	
Alcohol, n (%)				0.695
Yes	569 (68.80)	491 (69.06)	78 (67.24)	
No	258 (31.20)	220 (30.94)	38 (32.76)	
Hypertension, n (%)				0.460
Yes	366 (44.26)	311 (43.74)	55 (47.41)	
No	461 (55.74)	400 (56.26)	61 (52.59)	
Diabetes mellitus, n (%)				0.003
Yes	257 (31.08)	207 (29.11)	50 (43.10)	
No	570 (68.92)	504 (70.89)	66 (56.90)	
Metabolic Syndrome, n (%)				0.011
Yes	359 (43.41)	296 (41.63)	63 (54.31)	
No	468 (56.59)	415 (58.37)	53 (45.69)	
BMI (kg/m^2^)	30.10 (24.85, 35.80)	29.20 (24.50, 34.90)	34.55 (30.08, 39.92)	<0.001
WC (cm)	101.90 (88.55, 114.35)	100.80 (87.55, 112.60)	110.20 (100.12, 123.20)	<0.001
FPG (mg/dL)	96.00 (86.00, 117.50)	95.00 (85.00, 114.00)	102.00 (92.00, 151.00)	<0.001
HbA1c (%)	5.60 (5.30, 6.40)	5.60 (5.30, 6.25)	5.95 (5.50, 7.12)	<0.001
TC (mg/dL)	191.00 (165.00, 222.00)	190.00 (164.00, 220.00)	199.50 (172.00, 227.00)	0.032
TG (mg/dL)	139.00 (86.00, 223.00)	133.00 (81.50, 212.50)	179.50 (116.50, 273.50)	<0.001
HDL-c (mg/dL)	49.00 (40.00, 60.00)	49.00 (40.00, 60.00)	43.00 (38.00, 54.25)	0.005
Creatinine (mg/dL)	0.82 (0.67, 1.06)	0.83 (0.68, 1.07)	0.80 (0.66, 0.95)	0.136
BUN (mg/dL)	12.00 (10.00, 16.00)	12.00 (9.00, 16.00)	13.00 (10.00, 18.00)	0.090
Uric acid (mg/dL)	5.50 (4.40, 6.70)	5.40 (4.30, 6.65)	6.00 (5.00, 7.10)	<0.001
UACR (mg/g)	58.68 (36.74, 141.15)	57.03 (35.41, 130.19)	74.32 (41.82, 253.90)	0.004
eGFR (ml/min/1.73m^2^)	101.65 (80.53, 115.19)	101.07 (79.28, 114.99)	104.69 (85.48, 116.32)	0.314
TyG	8.85 (8.31, 9.46)	8.81 (8.25, 9.41)	9.22 (8.68, 9.79)	<0.001

Data are shown as median (25th, 75th percentiles) or percentages, *p*<0.05 considered statistically signiﬁcant.

CKD, Chronic kidney disease; BMI, Body mass index; WC, Waist circumference; FPG, Fasting plasma-glucose; HbA1c, Hemoglobin A1c; TC, Total cholesterol; TG, Triglyceride; HDL-c, High-density lipoprotein cholesterol; BUN, Blood urea nitrogen; UACR, Urinary albumin/creatinine ratio; eGFR, Estimated glomerular filtration rate; TyG, Triglyceride-glucose.

### 3.2 Baseline characteristics of CKD patients with or without sarcopenia in the Chinese cohort

Additionally, the study analyzed 1,038 patients with CKD in a Chinese cohort, comprising 860 individuals without sarcopenia and 178 with sarcopenia. The results demonstrated that patients in the sarcopenia group exhibited a markedly higher proportion of males than those in the non-sarcopenia group (76.40% vs. 56.16%, P< 0.001) and were significantly older (52.00 years vs. 44.00 years, P< 0.001). Regarding lifestyle habits, the prevalence of smoking was found to be significantly higher in the sarcopenia group than in the non-sarcopenia group (52.25% vs. 42.09%, P=0.013). However, no significant difference was observed in drinking habits (P=0.418). The prevalence of DM (34.83% vs. 24.30%, P=0.004) and metabolic syndrome (53.93% vs. 37.56%, P< 0.001) was significantly higher in the sarcopenia group than in the non-sarcopenia group. Furthermore, the sarcopenia group exhibited significantly elevated BMI, WC, FPG, HbA1c, TC, TG, and uric acid levels compared to the non-sarcopenia group (P< 0.05). Additionally, blood creatinine and BUN levels were significantly higher in the sarcopenia group than in the non-sarcopenia group (P values of 0.029 and 0.004, respectively). However, there was no significant difference in UACR and eGFR. TyG was significantly higher in the sarcopenia group (P< 0.001) (**Table 2**).

**Table 2:** Baseline characteristics of CKD patients with and without sarcopenia in the Chinese cohort.

Variables	Total (n=1038)	Non-sarcopenia (n=860)	Sarcopenia (n=178)	*P*
Gender, n (%)				<0.001
Male	619 (59.63)	483 (56.16)	136 (76.40)	
Female	419 (40.37)	377 (43.84)	42 (23.60)	
Age (years)	46.00 (34.00, 56.00)	44.00 (34.00, 55.00)	52.00 (38.00, 64.00)	<0.001
Marital Status, n (%)				0.141
Cohabitation	734 (70.71)	600 (69.77)	134 (75.28)	
Solitude	304 (29.29)	260 (30.23)	44 (24.72)	
Smoking, n (%)				0.013
Yes	455 (43.83)	362 (42.09)	93 (52.25)	
No	583 (56.17)	498 (57.91)	85 (47.75)	
Alcohol, n (%)				0.418
Yes	199 (19.17)	161 (18.72)	38 (21.35)	
No	839 (80.83)	699 (81.28)	140 (78.65)	
Hypertension, n (%)				0.278
Yes	645 (62.14)	528 (61.40)	117 (65.73)	
No	393 (37.86)	332 (38.60)	61 (34.27)	
Diabetes mellitus, n (%)				0.004
Yes	271 (26.11)	209 (24.30)	62 (34.83)	
No	767 (73.89)	651 (75.70)	116 (65.17)	
Metabolic Syndrome, n (%)				<0.001
Yes	419 (40.37)	323 (37.56)	96 (53.93)	
No	619 (59.63)	537 (62.44)	82 (46.07)	
BMI (kg/m^2^)	26.70 (24.40, 29.30)	26.40 (24.10, 28.83)	28.70 (26.10, 32.15)	<0.001
WC (cm)	93.55 (87.00, 100.80)	93.00 (86.27, 100.10)	97.10 (89.82, 106.00)	<0.001
FPG (mg/dL)	97.20 (91.80, 118.80)	97.20 (90.27, 113.40)	104.40 (95.40, 141.34)	<0.001
HbA1c (%)	5.30 (4.90, 6.50)	5.20 (4.90, 6.40)	5.80 (5.10, 7.18)	<0.001
TC (mg/dL)	176.38 (153.56, 202.20)	174.83 (152.79, 197.75)	186.24 (158.10, 215.16)	<0.001
TG (mg/dL)	121.38 (88.60, 189.60)	115.18 (85.06, 179.19)	152.39 (111.86, 232.57)	<0.001
HDL-c (mg/dL)	42.52 (36.33, 50.63)	42.90 (36.72, 51.02)	41.36 (35.17, 50.24)	0.074
Creatinine (mg/dL)	0.83 (0.70, 1.00)	0.82 (0.69, 1.00)	0.86 (0.74, 1.01)	0.029
BUN (mg/dL)	14.22 (11.76, 17.08)	14.03 (11.76, 16.80)	14.77 (13.00, 18.20)	0.004
Uric acid (mg/dL)	5.91 (4.80, 6.97)	5.82 (4.75, 6.87)	6.21 (5.20, 7.42)	0.002
UACR (mg/g)	65.50 (46.98, 88.38)	66.65 (47.19, 88.31)	60.43 (45.89, 89.85)	0.417
eGFR (ml/min/1.73m^2^)	101.26 (85.96, 114.99)	101.33 (86.66, 115.27)	100.81 (79.98, 113.18)	0.206
TyG	8.74 (8.35, 9.32)	8.65 (8.32, 9.27)	9.18 (8.68, 9.60)	<0.001

Data are shown as median (25th, 75th percentiles) or percentages, *p*<0.05 considered statistically signiﬁcant.

CKD, Chronic kidney disease; BMI, Body mass index; WC, Waist circumference; FPG, Fasting plasma-glucose; HbA1c, Hemoglobin A1c; TC, Total cholesterol; TG, Triglyceride; HDL-c, High-density lipoprotein cholesterol; BUN: Blood urea nitrogen; UACR, Urinary albumin/creatinine ratio; eGFR, Estimated glomerular filtration rate; TyG, Triglyceride-glucose.

### 3.3 Comparison of baseline characteristics between two cohorts

The proportion of males in the Chinese cohort (59.6%) was significantly higher than that in the US cohort (43.3%, P<0.001). Furthermore, Chinese participants were more likely to cohabit (70.7% vs. 56.8%, P<0.001). There was no statistically significant difference in smoking between the two cohorts (P=0.514). However, the prevalence of alcohol consumption was significantly lower in the Chinese cohort than in the US cohort (19.2% vs. 68.8%, P<0.001). The Chinese cohort exhibited a higher prevalence of hypertension (62.1% vs. 44.3%, P< 0.001) and a lower prevalence of diabetes (26.1% vs. 31.1%, P=0.018), while the prevalence of metabolic syndrome remained comparable (P=0.185). Moreover, Chinese participants exhibited significantly lower BMI, WC, HbA1c, TC, TG, and HDL-C levels compared to the US cohort (all P< 0.05), yet higher BUN and uric acid levels (P< 0.001). There were no significant differences between the two cohorts in the prevalence of sarcopenia (P=0.066) or TyG index (P=0.213) (**Supplementary Table S1**).

### 3.4 Relationship between TyG and sarcopenia in CKD patients

The relationship between TyG and sarcopenia was examined using three distinct models in the US cohort. The unadjusted model (Model 1) demonstrated a significant association between TyG and sarcopenia (OR 1.61, 95% CI 1.32-1.97, P< 0.001). After adjustment for gender and age (Model 2), the correlation exhibited a slight decline but remained statistically significant (OR 1.43, 95% CI 1.16-1.77, P< 0.001). Following further adjustment for marital status, smoking, alcohol consumption, hypertension, DM, metabolic syndrome, and eGFR (Model 3), the association between TyG and sarcopenia remained significant (OR 1.49, 95% CI 1.10-2.01, P=0.010). Furthermore, an analysis of TyG quartiles revealed a progressive increase in sarcopenia with elevated TyG levels. In comparison to the lowest TyG quartile, the risk of sarcopenia in the highest quartile was found to be 4.81 times higher (95% CI: 2.41-9.61, P< 0.001), 3.46 times higher (95% CI: 1.70-7.06, P< 0.001), and 4.01 times higher (95% CI: 1.66-9.66, P=0.002), respectively (**Table 3**).

**Table 3:** Relationship between TyG and **s**arcopenia in patients with CKD in the US cohort.

Variables	Model 1		Model 2		Model 3	
	OR (95%CI)	*P*	OR (95%CI)	*P*	OR (95%CI)	*P*
TyG	1.61 (1.32 ~ 1.97)	<0.001	1.43 (1.16 ~ 1.77)	<0.001	1.49 (1.10 ~ 2.01)	0.010
Categories						
Quartile 1	1.00 (Reference)		1.00 (Reference)		1.00 (Reference)	
Quartile 2	2.57 (1.24 ~ 5.36)	0.012	2.13 (1.01 ~ 4.48)	0.047	2.12 (1.01 ~ 4.52)	0.049
Quartile 3	3.63 (1.79 ~ 7.36)	<0.001	2.82 (1.37 ~ 5.81)	0.005	3.41 (1.53 ~ 7.60)	0.003
Quartile 4	4.81 (2.41 ~ 9.61)	<0.001	3.46 (1.70 ~ 7.06)	<0.001	4.01 (1.66 ~ 9.66)	0.002
*P* for trend		<0.001		<0.001		0.002

Model 1: crude.

Model 2: adjusted for Gender, Age.

Model 3: adjusted for Gender, Age, Marital status, Smoking, Alcohol, Hypertension, Diabetes mellitus, Metabolic syndrome, and eGFR.

TyG, Triglyceride-glucose; CKD, Chronic kidney disease; OR, Odds ratio; CI, Confidence interval; eGFR, Estimated glomerular filtration rate.

In the Chinese cohort, the relationship between TyG and sarcopenia in CKD patients was similarly analyzed. The results demonstrated a significant correlation between TyG and sarcopenia (OR 1.77, 95% CI 1.44-2.16, P< 0.001) in the unadjusted model (Model 1). In Model 2, the correlation decreased slightly (OR 1.60, 95% CI 1.30-1.97, P< 0.001) and remained significant (OR 1.62, 95% CI 1.20-2.18, P=0.002) after further adjustment for other confounders (Model 3). Similarly, analysis of TyG quartiles demonstrated that as the level of TyG increased, the risk of sarcopenia also increased. In comparison to the lowest TyG quartile, the risk of sarcopenia in the highest quartile was found to be increased by 4.32-fold (95% CI: 2.50-7.44, P< 0.001), 3.31-fold (95% CI: 1.89-5.80, P< 0.001), and 3.25-fold (95% CI: 1.64-6.46, P< 0.001), respectively. These findings further substantiate the role of TyG levels in predicting the risk of sarcopenia in patients with CKD, with consistent results across diverse geographical populations (**Table 4**).

**Table 4:** Relationship between TyG and **s**arcopenia in patients with CKD in the Chinese cohort.

Variables	Model 1		Model 2		Model 3	
	OR (95%CI)	*P*	OR (95%CI)	*P*	OR (95%CI)	*P*
TyG	1.77 (1.44 ~ 2.16)	<0.001	1.60 (1.30 ~ 1.97)	<0.001	1.62 (1.20 ~ 2.18)	0.002
Categories						
Quartile 1	1.00 (Reference)		1.00 (Reference)		1.00 (Reference)	
Quartile 2	1.85 (1.02 ~ 3.35)	0.042	1.68 (0.92 ~ 3.08)	0.092	1.64 (0.89 ~ 3.03)	0.115
Quartile 3	3.82 (2.21 ~ 6.62)	<0.001	3.30 (1.88 ~ 5.78)	<0.001	3.26 (1.78 ~ 5.97)	<0.001
Quartile 4	4.32 (2.50 ~ 7.44)	<0.001	3.31 (1.89 ~ 5.80)	<0.001	3.25 (1.64 ~ 6.46)	<0.001
*P* for trend		<0.001		<0.001		<0.001

Model 1: crude.

Model 2: adjusted for Gender, Age.

Model 3: adjusted for Gender, Age, Marital status, Smoking, Alcohol, Hypertension, Diabetes mellitus, Metabolic syndrome, and eGFR.

TyG, Triglyceride-glucose; CKD, Chronic kidney disease; OR, Odds ratio; CI, Confidence interval; eGFR, Estimated glomerular filtration rate.

### 3.5 RCS analysis

**Figure 2:**
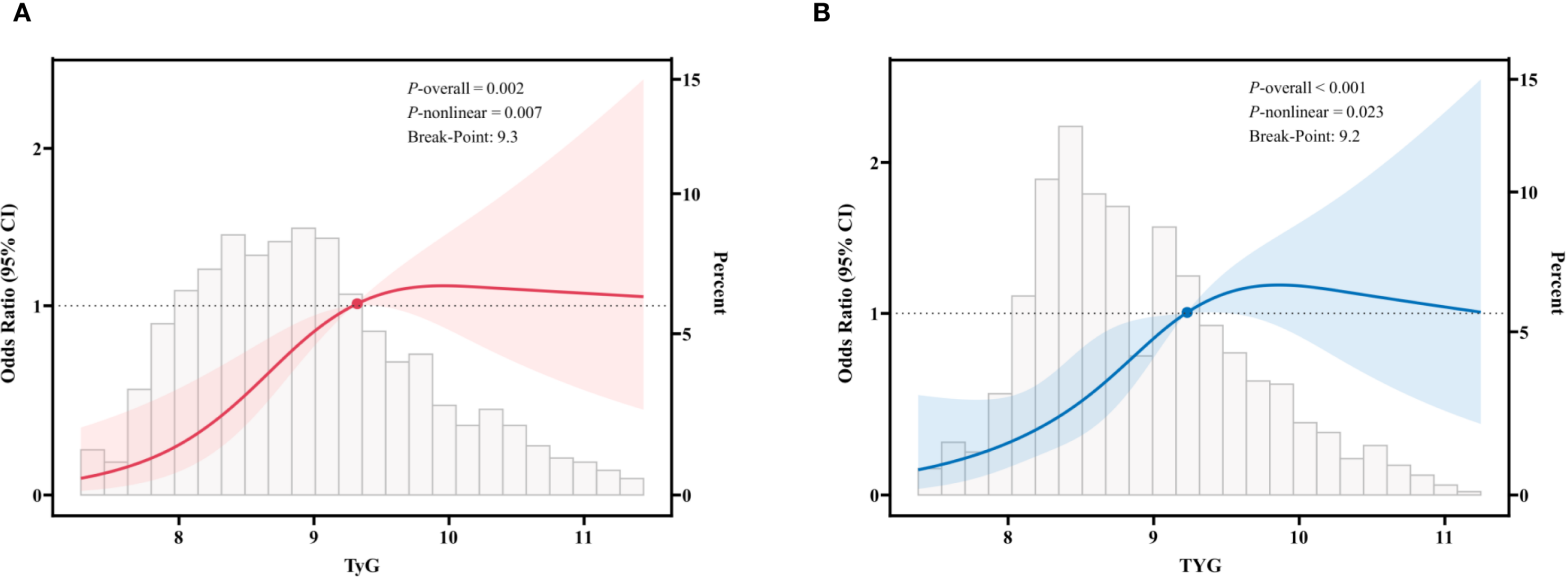
Restricted cubic spline fitting for the association between TyG and sarcopenia in patients with CKD. **(A)** in the US cohort; **(B)** in the Chinese cohort. The solid line displays the odds ratio, with the 95% CI represented by shading. They were adjusted for gender, age, marital status, smoking, alcohol, hypertension, diabetes mellitus, metabolic syndrome, and eGFR.

In this study, we employed restricted cubic spline fitting to analyze the relationship between TyG and sarcopenia in CKD patients, with assessments conducted separately for the US and Chinese cohorts. To more accurately assess the relationship, we adjusted for potential confounding variables, including gender, age, marital status, smoking, alcohol consumption, hypertension, DM, metabolic syndrome, and eGFR. The results demonstrated that in the US cohort (**Figure 2A**), there was a significant and positive association between TyG levels and sarcopenia, with a p-value of 0.002 and a nonlinear p-value of 0.007. Similarly, in the Chinese cohort (**Figure 2B**), there was a significant and positive association between TyG levels and sarcopenia, with a p-value of less than 0.001 and a nonlinear p-value of 0.023. The critical thresholds were determined to be 9.3 (US) and 9.2 (China), respectively. When the TyG index falls below these thresholds, a significant positive correlation with sarcopenia is indicated. However, beyond these thresholds, the risk growth rate slows down. This pattern is consistent across both cohorts, suggesting a saturation effect in the predictive value of the TyG index for sarcopenia risk.

### 3.6 Subgroup analysis

**Figure 3:**
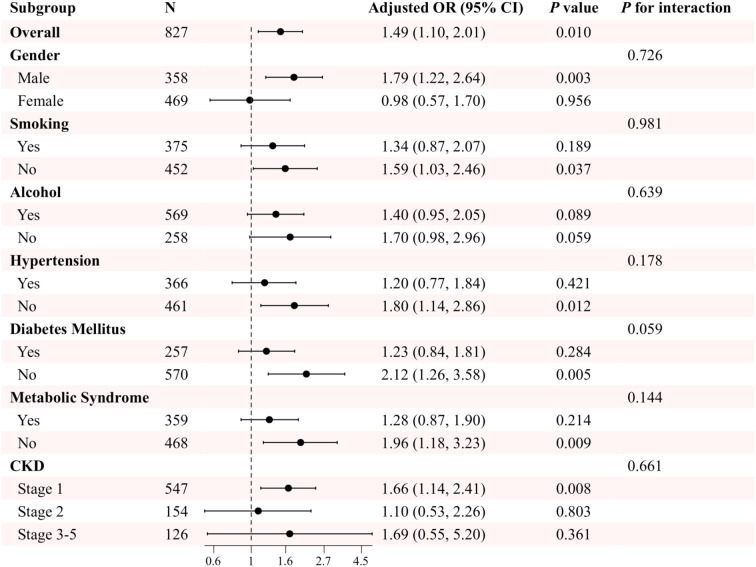
Subgroup analysis of the association between TyG and sarcopenia in patients with CKD in the US cohort. Adjusted variables: gender, age, marital status, smoking, alcohol, hypertension, diabetes mellitus, metabolic syndrome, and eGFR. The model was not adjusted for the stratification variables themselves in the corresponding stratification analysis.

In the US cohort, after adjusting for potential confounding variables, including gender, age, marital status, smoking and drinking habits, hypertension, DM, metabolic syndrome, and eGFR, the results demonstrated that in the overall CKD population, TyG levels were significantly and positively associated with sarcopenia, with an adjusted odds ratio of 1.49 (95% CI: 1.10, 2.01, P=0.010). Further subgroup analyses revealed that the association between TyG levels and sarcopenia in the different subgroups of gender, smoking, hypertension, DM, and metabolic syndrome exhibited some differences. For instance, the correlation between TyG levels and sarcopenia was more pronounced in the male cohort (OR=1.79, 95% CI: 1.22, 2.64, P=0.003), whereas no notable correlation was observed in the female cohort (OR=0.98, 95% CI: 0.57, 1.70, P=0.956). Furthermore, the correlation between TyG levels and sarcopenia was more pronounced in non-smokers, those without hypertension, those without diabetes, and those without metabolic syndrome (P< 0.05). Notably, no significant interactions were identified in any subgroup analyses, indicating that the observed association between TyG and sarcopenia remained consistent across all subgroups (P for interaction > 0.05) (**Figure 3**).

**Figure 4:**
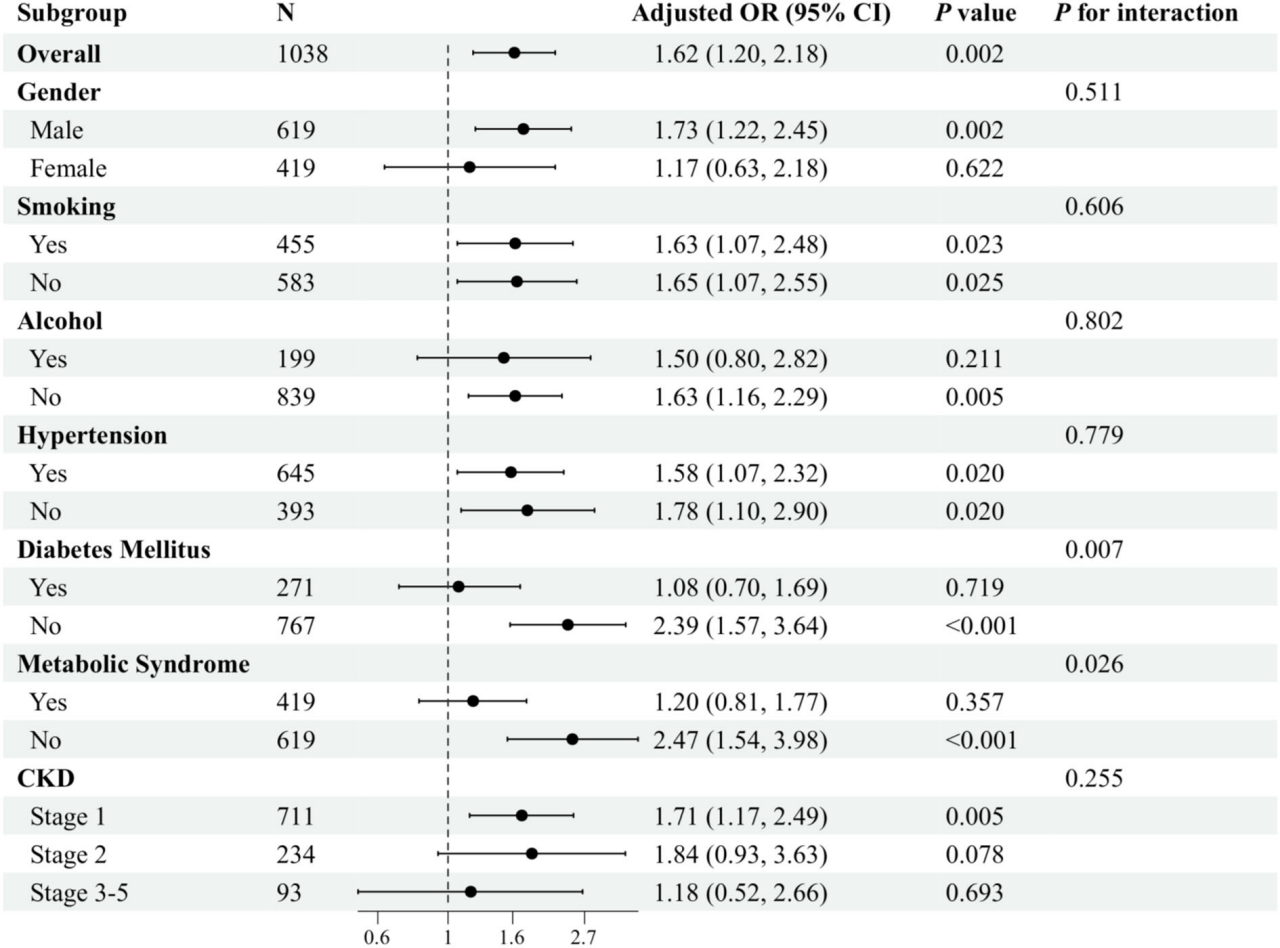
Subgroup analysis of the association between TyG and sarcopenia in patients with CKD in the Chinese cohort. Adjusted variables: gender, age, marital status, smoking, alcohol, hypertension, diabetes mellitus, metabolic syndrome, and eGFR. The model was not adjusted for the stratification variables themselves in the corresponding stratification analysis.

In the Chinese cohort, the results of the subgroup analyses further demonstrated this association’s variability across populations after adjusting for various potential confounding variables. In the smoking and hypertension subgroups, TyG levels were significantly associated with sarcopenia regardless of whether or not the subjects smoked and irrespective of whether or not they had hypertension (P< 0.05). Furthermore, no significant interactions were found between the groups (P for interaction > 0.05). In both the gender and the drinking subgroups, TyG levels were significantly associated with sarcopenia in both males and non-drinkers (P< 0.05). Conversely, no significant associations were observed in females and the drinking subgroups. However, no significant interactions were found between groups (P for interaction > 0.05). Notably, significant interactions were observed in the subgroup analyses of patients with DM and metabolic syndrome (P for interaction< 0.05). The association between TyG levels and sarcopenia was more pronounced in patients without DM and in patients without metabolic syndrome (P< 0.001) (**Figure 4**).

## 4 Discussion

This study aimed to investigate the association between the TyG index and sarcopenia. To this end, data from two cohorts of CKD patients from the United States and China were analyzed. The findings indicated that the TyG index demonstrated a notable and consistent nonlinear positive correlation with sarcopenia in CKD patients in the US and Chinese cohorts. This association remained consistent across gender, lifestyle habits, and chronic disease background.

The present study provides further evidence to support the notion that insulin resistance plays a pivotal role in the pathogenesis of sarcopenia. The TyG index, a straightforward and readily available metabolic index, effectively reflects the body’s insulin resistance status by integrating two conventional biochemical indexes: triglycerides and fasting blood glucose (13). The findings of this study demonstrate a high degree of concordance with prior research, indicating that a one-unit increase in the TyG index is associated with a 54–61% increase in the risk of sarcopenia. This observation aligns closely with the results reported in the general population, thereby substantiating the universality of the TyG index as a reliable predictive marker (17, 18). It can thus be hypothesized that insulin resistance may also be involved in the mechanism of the onset and development of sarcopenia in CKD patients.

Insulin resistance is a prevalent phenomenon among CKD patients, with a complex and varied pathogenesis. Multiple factors, including impaired post-insulin receptor signaling, poor dietary habits, physical inactivity, metabolic acidosis, anemia, vitamin D insufficiency, chronic inflammation, oxidative stress, and adipokine imbalance, influence it (25–28). These factors are intertwined and collectively contribute to substantially reducing insulin sensitivity in patients with CKD. To maintain glycemic equilibrium, the body must compensate for the increase in insulin synthesis and secretion, which induces hyperinsulinemia. As CKD progresses, the prevalence of insulin resistance increases (25).

There is a robust correlation between insulin resistance and sarcopenia in patients with CKD. Insulin resistance affects muscle mass and function in patients with CKD through a variety of mechanisms, thereby increasing their risk of developing sarcopenia. In insulin resistance, the promotion of protein synthesis by insulin is impaired, whereas its promotion of protein catabolism is enhanced. This results in a state of disequilibrium between protein synthesis and catabolism in CKD patients, with a reduction in muscle protein synthesis and an acceleration in catabolism, ultimately leading to sarcopenia development (19, 20). Furthermore, the inflammation and oxidative stress levels within the body are markedly elevated in the context of insulin resistance. These pathological processes further impair muscle cell function and structure and accelerate the loss of muscle mass (19, 29). It is noteworthy that patients with CKD are frequently associated with metabolic acidosis, which represents an additional critical factor influencing insulin resistance and the development of sarcopenia. Metabolic acidosis has been demonstrated to inhibit muscle anabolism and promote catabolism, thereby exacerbating the onset and progression of sarcopenia (19, 30). Furthermore, the accumulation of uremic toxins in patients with CKD has been demonstrated to have a deleterious impact on muscle mass and function. These toxins may also contribute to the onset and development of sarcopenia by interfering with insulin signaling, promoting inflammation and oxidative stress, and other mechanisms (27, 31). In conclusion, the association between insulin resistance and sarcopenia in patients with CKD may result from a complex interplay between multiple interrelated and interacting mechanisms, collectively influencing muscle health and overall health in patients with CKD.

The findings of this study offer novel insights into the prevention and treatment of complications associated with sarcopenia in patients with CKD. As a prevalent and significant complication among patients with CKD, sarcopenia markedly impacts the quality of life and prognosis of these patients. At present, the treatment of sarcopenia in patients with CKD remains inadequate, with nutritional supplementation and exercise rehabilitation training representing the primary modalities employed (32, 33). The findings of this study indicate that sarcopenia in CKD patients may be mitigated by modifying the TyG index. Specifically, dietary optimization and lifestyle modification to reduce triglyceride and fasting blood glucose levels, decreasing the TyG index, may prove an effective strategy for preventing and treating sarcopenia in CKD patients.

Furthermore, the present study demonstrated that the correlation between TyG and the likelihood of developing sarcopenia exhibited some degree of variability when subjected to different subgroup analyses. In particular, a significant interaction effect was observed in the subgroup analysis of diabetes and metabolic syndrome in the Chinese cohort. The correlation between TyG level and sarcopenia was more pronounced in the subgroups of patients who did not have diabetes and those who did not have metabolic syndrome. This phenomenon may be attributed to the fact that there were fewer potential confounding variables in participants without these disorders compared with individuals with diabetes or metabolic syndrome, which may have resulted in a more pronounced and significant association between TyG levels and sarcopenia. Additionally, the complex interactions between diseases such as DM and metabolic syndrome with CKD and sarcopenia may obscure the direct link between TyG and sarcopenia (34). These findings indicate that differentiated intervention strategies may be required in clinical practice for CKD patient groups with different characteristics to reduce the risk of sarcopenia effectively. The disparities in interaction effects observed between the Chinese and US cohorts in the diabetes and metabolic syndrome subgroups are primarily attributable to the combined influence of disease management strategies, metabolic phenotype characteristics, and the progression of chronic kidney disease. In the US cohort, the rate of insulin use among diabetes patients was significantly higher, and this treatment approach may weaken the predictive efficacy of the TyG index for sarcopenia by improving insulin resistance. Conversely, Chinese diabetes patients predominantly depend on oral hypoglycemic agents, and persistent insulin resistance serves to reinforce the TyG index’s capacity to emit a more pronounced risk warning signal. Furthermore, patients diagnosed with metabolic syndrome in the US predominantly manifest abdominal obesity as the predominant phenotype. Obesity itself, a significant confounding factor for sarcopenia, serves to attenuate the effect of the TyG index. Conversely, Chinese patients with a similar condition predominantly exhibit elevated TG levels and FPG concentrations, which closely align with the fundamental components of the TyG index. Of particular significance is the observation that the Chinese cohort exhibits a higher prevalence of advanced CKD, with uremic toxin accumulation exerting its influence on TyG’s predictive specificity through pathways independent of insulin resistance. In light of these factors, the TyG index exhibits augmented predictive value for sarcopenia in populations with CKD and low prevalence of insulin therapy, where abdominal obesity is less pronounced. Future research endeavors should integrate medication history and toxin accumulation indicators to develop precise stratification models.

The value of this study lies in its integration of data from two cohorts, one from the United States and one from China, which allows for a cross-regional and cross-cultural perspective that significantly enhances the generalizability and reliability of the findings. By adopting uniform inclusion and exclusion criteria, the study ensured the homogeneity of the study population. Furthermore, a multivariate adjustment model was employed to effectively control potential confounders, thus improving the accuracy of the study findings. Nevertheless, this study is not without limitations. Firstly, it was impossible to establish causality due to the cross-sectional study design. Secondly, the study population was limited to CKD patients in the United States and China, which may not fully reflect the global status of CKD patients. Moreover, despite the adjustment for multiple potential confounding variables, there may still be unmeasured confounding factors (e.g., genetic background, environmental factors, etc.) that could influence the study results. In light of these considerations, future studies should adopt a prospective design to validate the causal relationship between TyG and the risk of sarcopenia further. Furthermore, the sample size should be expanded to include data from CKD patients in more countries and regions, thus enhancing the generalizability and accuracy of the study results. Furthermore, it is essential to investigate the influence of potential confounding factors on the study outcomes to develop a more robust study design, thereby comprehensively elucidating the association between the TyG index and the risk of sarcopenia.

## 5 Conclusions

In conclusion, this study demonstrated a notable correlation between the TyG index and the likelihood of developing sarcopenia in patients with CKD by examining data from many subjects. This finding offers novel insights and avenues for clinical diagnosis and treatment, as well as valuable references and lessons for future research. In future studies, it would be beneficial to explore further the specific mechanism of action between the TyG index and sarcopenia in CKD patients. Additionally, personalized treatment plans for different subgroups of patients could be developed to promote the advancement of sarcopenia prevention and treatment in CKD patients.

## Data Availability

The original contributions presented in the study are included in the article/**Supplementary Material**. Further inquiries can be directed to the corresponding author.
