# Supplementary material for: Association between triglyceride-glucose index and sarcopenia in patients with chronic kidney disease
Source: Front Endocrinol (Lausanne). 2025 Sep 18;16:1626241. doi: 10.3389/fendo.2025.1626241 (PMC12488414; doi:10.3389/fendo.2025.1626241)
Supplement: Supplementary file 1 [file Table1.docx]

### TABLE S1 Patient demographics and baseline characteristics.

| **Variables** | **US cohort (n = 827)** | **Chinese cohort (n = 1038)** | ***P*** |
| --- | --- | --- | --- |
|  |  |  |  |
| Gender, n (%) |  |  | <0.001 |
| Male | 358 (43.3%) | 619 (59.6%) |  |
| Female | 469 (56.7%) | 419 (40.4%) |  |
| Age (years) | 46 (35, 53) | 46 (34, 56) | 0.001 |
| Marital Status, n (%) |  |  | <0.001 |
| Cohabitation | 470 (56.8%) | 734 (70.7%) |  |
| Solitude | 357 (43.2%) | 304 (29.3%) |  |
| Smoking, n (%) |  |  | 0.514 |
| Yes | 375 (45.3%) | 455 (43.8%) |  |
| No | 452 (54.7%) | 583 (56.2%) |  |
| Alcohol, n (%) |  |  | <0.001 |
| Yes | 569 (68.8%) | 199 (19.2%) |  |
| No | 258 (31.2%) | 839 (80.8%) |  |
| Hypertension, n (%) |  |  | <0.001 |
| Yes | 366 (44.3%) | 645 (62.1%) |  |
| No | 461 (55.7%) | 393 (37.9%) |  |
| Diabetes mellitus, n (%) |  |  | 0.018 |
| Yes | 257 (31.1%) | 271 (26.1%) |  |
| No | 570 (68.9%) | 767 (73.9%) |  |
| Metabolic Syndrome, n (%) |  |  | 0.185 |
| Yes | 359 (43.4%) | 419 (40.4%) |  |
| No | 468 (56.6%) | 619 (59.6%) |  |
| BMI (kg/m^2^) | 30 (25, 36) | 27 (24, 29) | <0.001 |
| WC (cm) | 102 (89, 114) | 94 (87, 101) | <0.001 |
| FPG (mg/dL) | 96 (86, 118) | 97 (92, 119) | <0.001 |
| HbA1c (%) | 5.60 (5.30, 6.40) | 5.30 (4.90, 6.50) | <0.001 |
| TC (mg/dL) | 191 (165, 222) | 176 (154, 202) | <0.001 |
| TG (mg/dL) | 139 (86, 224) | 121 (89, 190) | 0.012 |
| HDL-c (mg/dL) | 49 (40, 60) | 43 (36, 51) | <0.001 |
| Creatinine (mg/dL) | 0.82 (0.67, 1.06) | 0.83 (0.70, 1.00) | 0.709 |
| BUN (mg/dL) | 12.0 (10.0, 16.0) | 14.2 (11.8, 17.1) | <0.001 |
| Uric acid (mg/dL) | 5.50 (4.40, 6.70) | 5.91 (4.80, 6.97) | <0.001 |
| UACR (mg/g) | 59 (37, 141) | 65 (47, 88) | 0.470 |
| eGFR (ml/min/1.73m^2^) | 102 (81, 115) | 101 (86, 115) | 0.312 |
| TyG | 8.85 (8.30, 9.47) | 8.74 (8.35, 9.32) | 0.213 |
| Sarcopenia, n (%) |  |  | 0.066 |
| Yes | 116 (14.0%) | 178 (17.1%) |  |
| No | 711 (86.0%) | 860 (82.9%) |  |
